# Lytic HSV-1 infection induces the multifunctional transcription factor Early Growth Response-1 (EGR-1) in rabbit corneal cells

**DOI:** 10.1186/1743-422X-8-262

**Published:** 2011-05-27

**Authors:** Gautam R Bedadala, Jayavardhana R Palem, Lorna Graham, James M Hill, Harris E McFerrin, Shao-Chung Hsia

**Affiliations:** 1 Department of Pharmaceutical Sciences, University of Maryland Eastern Shore School of Pharmacy, Princess Anne, MD, USA; 2 Department of Basic Pharmaceutical Sciences, University of Louisiana Monroe School of Pharmacy, Monroe, LA, USA; 3 Department of Ophthalmology, Louisiana State University Health Sciences Center, New Orleans, LA, USA; 4 Department of Biology, Xavier University of Louisiana, New Orleans, LA, USA

**Keywords:** Egr-1, HSV-1, lytic infection

## Abstract

**Background:**

Herpes simplex virus type-1 (HSV-1) infections can cause a number of diseases ranging from simple cold sores to dangerous keratitis and lethal encephalitis. The interaction between virus and host cells, critical for viral replication, is being extensively investigated by many laboratories. In this study, we tested the hypothesis that HSV-1 lytic infection triggers the expression of important multi-functional transcription factor Egr1. The mechanisms of induction are mediated, at least in part, by signaling pathways such as NFκB and CREB.

**Methods:**

SIRC, VERO, and 293HEK cell lines were infected with HSV-1, and the Egr-1 transcript and protein were detected by RT-PCR and Western blot, respectively. The localization and expression profile of Egr-1 were investigated further by immunofluorescence microscopy analyses. The recruitment of transcription factors to the Egr-1 promoter during infection was studied by chromatin immunoprecipitation (ChIP). Various inhibitors and dominant-negative mutant were used to assess the mechanisms of Egr-1 induction and their effects were addressed by immunofluorescence microscopy.

**Results:**

Western blot analyses showed that Egr-1 was absent in uninfected cells; however, the protein was detected 24-72 hours post treatment, and the response was directly proportional to the titer of the virus used for infection. Using recombinant HSV-1 expressing EGFP, Egr-1 was detected only in the infected cells. ChIP assays demonstrated that NFкB and cAMP response element binding protein (CREB) were recruited to the Egr-1 promoter upon infection. Additional studies showed that inhibitors of NFкB and dominant-negative CREB repressed the Egr-1 induction by HSV-1 infection.

**Conclusion:**

Collectively, these results demonstrate that Egr-1 is expressed rapidly upon HSV-1 infection and that this novel induction could be due to the NFкB/CREB-mediated transactivation. Egr-1 induction might play a key role in the viral gene expression, replication, inflammation, and the disease progression.

## Background

Herpes simplex virus type-1 (HSV-1) is a common pathogen with worldwide seroprevalence rates ranging from 50% to 90% [1-3]. It is a neurotropic virus that is in the subfamily of *alpha herpesvirinae*. Initial or primary infection with HSV-1 mostly occurs during childhood in mucoepithelial surfaces and is generally mild or asymptomatic. Upon infection of epithelial cells, HSV-1 initiates lytic replication, and at the end of this cycle, the virus infects sensory neurons proximal to the site of primary infection. Virions can travel via retrograde axonal transport to the cell bodies of neurons in the trigeminal ganglia where lifelong latency is established [4]. The latent virions can reactivate due to unknown causes and prohibit subsequent lytic infections. The most common form of lytic infection is the cold sore or fever blister where viral replication takes place in the orofacial mucosa [5]. Infection of the brain leads to herpes encephalitis, a dangerous condition that can cause permanent neurological damage with high mortality [5]. Orofacial infection, although less threatening, represents the major route for transmission to a naive host.

In addition to encephalopathy and orofacial infection, HSV-1 also infects the eyes leading to 8 cases of herpetic keratitis per 100,000 population every year [6]. Keratitis refers to inflammation of corneal epithelium due to a number of irritants such as infections by viruses, bacteria, and fungi. Herpetic keratitis is the major cause of corneal blindness, with unilateral visual impairment occurring in at least one-third of patients with recurrent disease [7].

Lytic infections of HSV-1 are typically characterized by the entry of virus into the cell and translocation of the viral genome into the nucleus followed by replication and evasion of host defenses in order to disperse and persist in the environment. Gene expression of HSV-1 is tightly regulated in a cascade fashion. The three temporal classes of genes are designated immediate-early (α), early (β) and late (γ) genes [8]. There are five α genes, designated ICP0, ICP4, ICP22, ICP27, and ICP47. The α genes were defined by the presence of the cis element for induction by VP16, which interacts with cellular factors, including the protein Oct-1, a homeobox protein, to activate viral immediate early (IE) gene transcription in trans [9,10]. The expression of β genes requires the expression of α genes, especially functional ICP4. ICP0 enhances the ability of ICP4 to trans-activate γ genes [11]. The expression of γ gene has been shown to be blocked by effective concentrations of DNA synthesis inhibitors [12]. All of these steps require interaction between viral and host components. The communication between virus and host factors is being investigated by many laboratories but is not understood completely, and identification of host factors that interact with the virus remains a challenge. Additional information will identify new drug targets for treatment and will aid in understanding the complex processes of initiation of HSV-1 latency and reactivation.

Our laboratory examined the hypothesis that HSV-1 infection of epithelial cells including a cell line from rabbit cornea rapidly induced the expression of the cellular transcription factor early growth response-1 (Egr-1), also known as NGFI-a, Zif268, Krox24, TIS8, and ZENK. The Early Growth Response (EGR) family belongs to the C2H2-type zinc-finger proteins [13-15]. Egr-1 functions as a convergence point for many signaling cascades and is known to play an important role in regulating inflammation, cell proliferation, and apoptosis [16]. Our previous transient cotransfection studies with promoter plasmids and Egr-1 expression vector showed that Egr-1 regulated HSV-1 ICP22 and ICP4 promoter activity [17]. In the present study, we demonstrate for the first time that Egr-1 protein is induced rapidly in cells such as SIRC and VERO upon infection. Using inhibitors of the transcription factors CREB and NFκB, we further investigate the mechanism by which HSV-1 induces Egr-1 activation

## Methods

### Cells, viruses and culture conditions

VERO (derived from kidney epithelial cells of the African Green Monkey) cells and HEK293 cells were grown in DMEM supplemented with 10% FBS. SIRC (Rabbit cornea) cells obtained from ATCC (Cat#: CCL-60) were grown in MEM supplemented with 10% FBS. The RE-EGFP strain of HSV-1 was used throughout the study [18]. Unless otherwise indicated, all viral infections were carried with a MOI of 5 for 1 hour.

### Western Blot analysis

Protein extract was subjected to 10% sodium dodecyl sulfate polyacrylamide gel electrophoresis and transferred onto nitrocellulose membranes. The blots were blocked using PBS with 5% (wt/vol) non-fat dry milk and washed in PBS. Rabbit anti-Egr-1 polyclonal antibody (Santa Cruz SC-110x) or cAMP response element binding (CREB) Ser-133 phosphorylation (Abcam#ab32096) was used at a dilution of 1:1,000. Anti-a-Tubulin mouse antibody (Calbiochem, Cat#: CP06, San Diego, CA) was added at a dilution of 1:10,000. The chemiluminiscent signal from the membranes was detected by Syngene GeneGnome HR Bioimaging system (Frederick, MD). The protocol was performed essentially as described by the manufacturer.

### Immunofluoroscence

Approximately 20,000 cells were placed in a multi-chamber slide (Cat# 354104 BD Falcon) with respective media one day before infection. The cells were pretreated for 3 hours with inhibitors Bay 11-7082 (Calbiochem#196871) and NBD binding peptide (Calbiochem Cat#: 480025) for the NFкB inhibition study. After infection cells were rinsed once with 2 ml PBS for 5 min and fixed with 100% methanol at -20°C. Slides were incubated with 2% normal blocking serum followed by incubation with primary antibody for Egr-1 (Millipore# MAB10073) overnight at 4°C. The slides were then incubated with fluorescent conjugated secondary antibody (Invitrogen cat# A21424), at RT for 1 hr. Finally, the slides were mounted with fluorescent mounting medium containing DAPI. The expression of enhanced green fluorescent protein (EGFP), red fluorescence, and DAPI staining were accessed by an Olympus fluorescence microscope (IX71) coupled with an Olympus digital camera photo apparatus (DP71). Imaging analysis was performed by using Olympus DP controller software.

### Reverse transcriptase PCR (RT-PCR)

Total RNA was extracted from the cells using trizol reagent (Invitrogen). RT-PCR was performed using Superscript One-Step RT-PCR (Invitrogen) with 0.5 μg of total RNA and primer set for reaction. Their sequences are as follows: Egr-1: 5'-AGA CCA GTT ACC CCA GCC AAA C-3'and 5'-AAA ATG TCA GTG TTC GGC GTG-3'; EGFP: 5'-GCA GAA GAA CGG CAT CAA GGT G-3' and 5'-TGG GTG CTC AGG TAG TGG TTG TC-3'. The reverse transcription/PCR reaction was carried out at 45°C for 20 min followed by 25 cycles of 94°C for 30 s, 55°C for 30 s, and 68°C for 30 s. The RT-PCR products were analyzed by 2% agarose gel electrophoresis.

### Chromatin Immunoprecipitation (ChIP) assays

Cell monolayers were treated with 1% formaldehyde solution for 10 min at room temperature. Cells were then harvested and subjected to sonication. The lysed samples were centrifuged for 10 min at 13,000 rpm at 4°C and the supernatant was diluted 10-fold with RIPA buffer containing protease inhibitor. Immunoprecipitation was then performed with Dynabeads Protein A (Invitrogen, Cat#: 100.01D) with antibodies against CREB (Abcam#ab32096) and NFкB (Abcam#ab7970). To analyze immunoprecipitated DNA, PCR amplification was performed with primers against Egr-1 promoter: 5'-TGG GGG GCT TCA CGT CAC TC-3' and 5'-AAG TTC TGC GGC TGG ATC TCT C-3''. The products were analyzed by 2% agarose gel electrophoresis.

### Transfection studies involving the dominant negative mutants of CREB

Approximately 20,000 cells were placed in a multichamber slide (Cat# 354104 BD Falcon) with respective media one day before transfection. The cells were cotransfected with phMGFP (Promega Cat #E6421) and one of the vectors (pCMV-CREB133 or pCMV-KCREB) from the CREB dominant negative vector set (Clonetech Cat# 631925) using lipofectamine (Invitrogen). fter 2 days, the cells were infected with virus (MOI = 1) for 1 hour. The cells were then incubated in fresh medium for 24 hours. Immunofluorescence was carried out using anti-Egr-1 antibody as described above.

## Results

### Egr-1 protein is induced by HSV-1 infection

**Figure 1:**
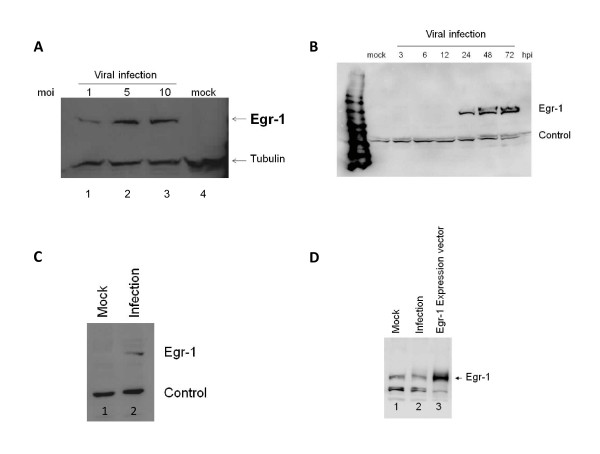
**Egr-1 protein was induced by HSV-1 infection 24 hours post treatment**. a.) This figure is a western blot analysis showing the expression of Egr-1 protein (83 Kda) in infected VERO cells 24 hours post treatment. MOI of 1, 5, and 10 were used and more viruses led to more Egr-1 production. Noted that infection at higher moi (Lane 3) led to cell death therefore no significant increase of Egr-1 production was observed. b.) Egr-1 was induced by HSV-1 infection (MOI-5) in VERO cells and detectable levels were observed at 24, 48, and 72 hours post treatment. c.) Egr-1 induction was also seen in SIRC cells infected with a MOI of 5. d.) Uninfected HEK 293 cells express Egr1 (lane 1), and no induction was seen upon infection with HSV-1 MOI 10 (lane 2). Overexpression of Egr-1 was seen in HEK293 cells transfected with Egr1 expression vector (lane 3).

**Figure 2:**
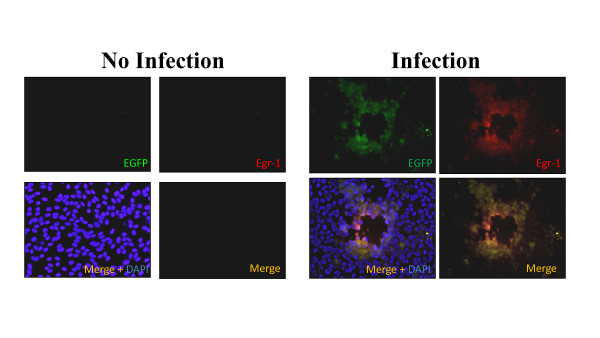
**Immunofluorescence microscopy indicates expression of Egr-1 in HSV-1 infected cells**. Immunofluorescence data indicated that Egr-1(red) is expressed only in the cells infected with HSV-1 EGFP virus (green). Infection controls showed no EGFP signal or Egr-1, indicating the specificity of the antibody.

The expression of Egr1 was established first using the VERO cell line as the model for our study because HSV-1 lytic gene expression is well characterized in these cells [19-23]. Western blotting analysis showed that Egr-1 protein was not present in VERO cells but was induced upon infection and that its induction was enhanced with increased viral infection (Figure 1A). A time-course study revealed that detectable levels of Egr-1 protein were present at 24 hours post infection, and similar levels were detected at 48 and 72 hours post infection (Figure 1B). Our data also indicated that Egr-1 protein was induced in rabbit corneal cells SIRC under similar conditions (Figure 1C). In addition, human corneal cell line HCE-2 was sufficient to produce Egr-1 upon HSV-1 infection (data not shown). However, Egr-1 was not inducible in HEK293 cells. Endogenous Egr-1 was present in uninfected 293HEK cells and the production was not stimulated by viral infection (Figure 1D). To further confirm the induction of Egr-1 by HSV-1, immunofluorescence staining was performed on infected cells. A recombinant HSV-1 constitutively expressing EGFP was used to infect the cells followed by fixation, blocking, and incubation with primary and secondary Egr-1 antibody (shown in red). The results supported the previous observation that Egr-1 protein (red) was co-expressed only in the cells infected with EGFP virus (green) (Figure 2). Together, these results strongly indicated that Egr1 protein was induced by HSV-1 infection.

### Egr-1 mRNA was detected at 1 hour post infection

**Figure 3:**
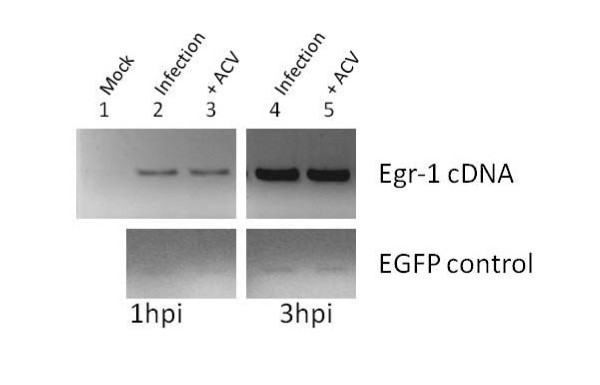
**Egr-1 mRNA was detected at 1 hour post treatment**. VERO cells were infected with HSV-1 BE EGFP virus or with UV-inactivated virus at a MOI of 5. Total mRNA was isolated at 1, 2,3,12, and 24 hours post treatment and RT-PCR was performed.

RT-PCR assays were used to analyze Egr-1 mRNA transcript expression upon infection. Interestingly, results showed that Egr-1 mRNA was produced as early as 1 hour post infection and a higher concentration of Egr-1 mRNA was observed 3 hours post infection (Figure 3). To address whether viral replication was necessary for Egr-1 production, the same analyses were conducted in the presence of acyclovir (ACV, 50 μM); the results indicated that viral replication was not required for Egr-1 mRNA expression (Figure 3). These results demonstrated that HSV-1 infection increased Egr1 mRNA transcript expression which occurred immediately after viral inoculation.

### NFкB and pCREB were recruited to the Egr1 promoter upon infection

**Figure 4:**
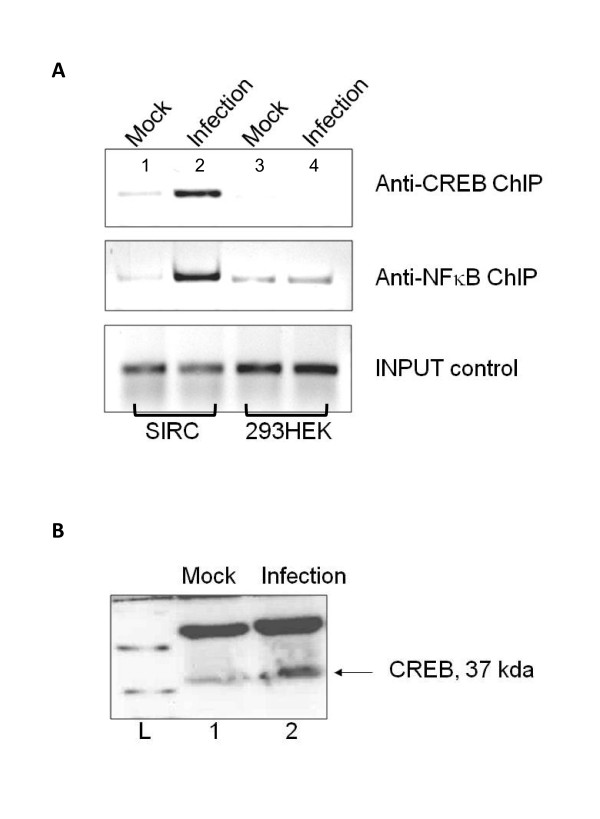
**NFкB and CREB were recruited to the Egr-1 promoter upon infection**. a.) Chip assay was performed using antibodies against NFкB and CREB. Lane1: Noninfected SIRC cells. Lane 2: SIRC cells infected with HSV-1 at a MOI of 5. Lane 3: HEK293 cells infected with virus at a MOI of 5. PCR was performed using primers for the Egr-1 promoter. For comparison, input controls (lanes 1-3) indicated the PCR products from samples prior to immunoprecipitation. b.) Over-expression of CREB protein (37 kda) was seen in VERO cells infected with HSV-1 (lane 2) when compared to the mock-infected control (lane 1).

The Egr-1 promoter exhibits several transcription factor binding sites such as NFкB [24] and CREB [25,26]. NFкB has been shown to interact with Egr-1 promoter [27,28]. ChIP assays were performed 24 hours post infection to examine the recruitment of NFкB and pCREB to the Egr1 promoter by HSV-1 infection. The results indicated that NFкB and pCREB were recruited to the Egr-1 promoter upon infection in SIRC cells (compare Figure 4A, lanes 1 and 2). This type of recruitment was not seen in HEK293 cells, which exhibited no Egr1 induction upon infection (compare Figure 4A, lanes 3 and 4). Additional infection experiments showed increase in pCREB protein level during HSV-1 infection (Figure 4B, lane 2). These observations suggested that NFκB and pCREB could play roles in regulating HSV-1-mediated Egr1 expression.

### Dominant-negative CREB decreased the Egr-1 induction observed upon infection

**Figure 5:**
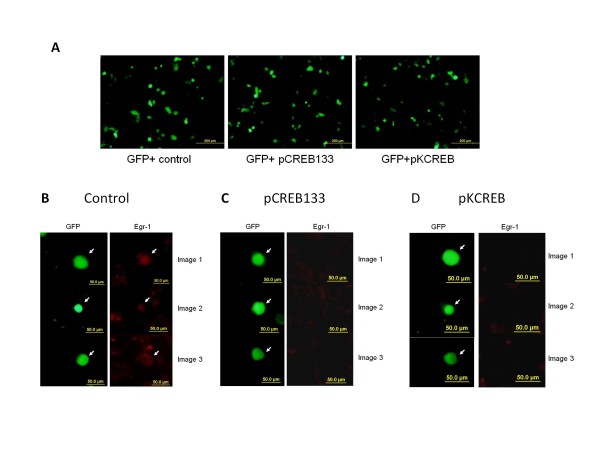
**Dominant negative CREB inhibited the induction of Egr-1 upon HSV-1 infection**. VERO cells were transfected with phMGFP alone and in combination with CREB dominant negative vectors pCREB133 or pKCREB. a.) The transfection efficiency of the three groups is similar as shown by the number of GFP-positive cells. b.) Cells infected with a control vector show induction of Egr-1 protein (red) whereas cells infected with the dominant negative mutants, CREB133(c) or KCREB (d) do not show the induction of the Egr-1 protein.

Immunofluorescent analyses were performed to investigate if CREB participated in Egr1 induction. Following transfection with two dominant negative isoforms of CREB, pCREB 133 or pKCREB, the number of green fluorescent cells observed was similar after 2 days, indicating that transfection efficiency was equivalent (Figure 5A). Additional data showed that the use of dominant negative CREB inhibited the induction of Egr-1 (Figure 5C and 5D) when compared to the GFP control (Figure 5B). Plasmid pCMV-CREB133 encodes a mutated form of CREB, blocking cAMP-induced transcription, and preventing CREB phosphorylation at S133. Plasmid pCMV-KCREB also encodes a mutated form of CREB, blocking cAMP-induced transcription. These results indicated that CREB was involved in the virus-mediated Egr1 expression.

### NFкB inhibition reduced Egr-1 expression by infection

**Figure 6:**
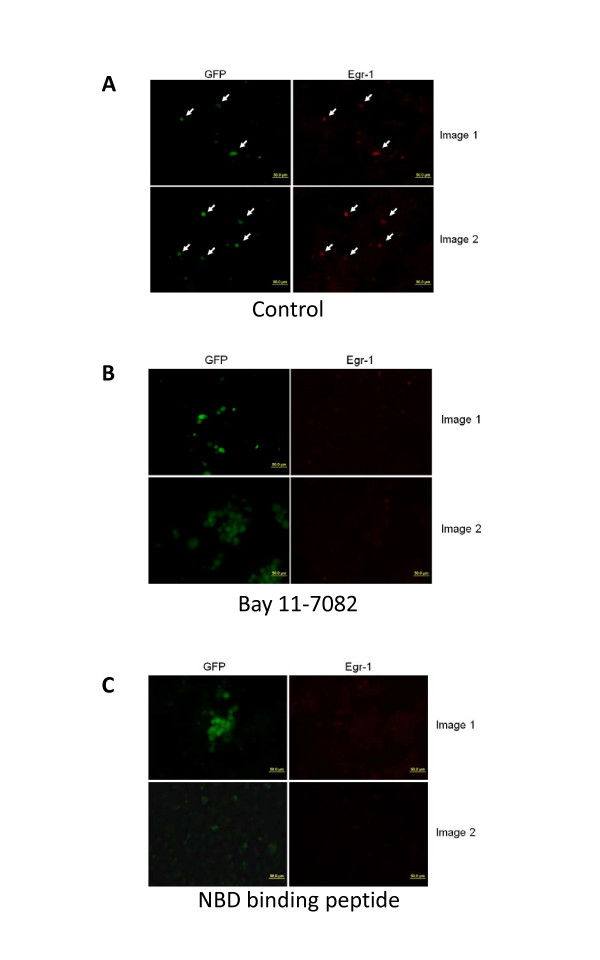
**NFкB inhibitors abolished the induction of Egr-1 upon infection**. Egr-1 expression was not induced in cells pretreated with 1 μM of BAY 11-7082 (b) or 10 μM NBD binding peptide (c) after infection with HSV-1 compared the control (a)

The NFкB inhibitor Bay11-7082 (Calbiochem, San Diego, CA) specifically targets the TNF-α-inducible phosphorylation of IκBα, irreversibly inhibiting and decreasing expression of NFκB [29]. NBD binding peptide is a cell-permeable fusion peptide that inhibits cytokine-induced NF-κB activation and NF-κB-dependent gene expression [30]. Immunofluorescence studies showed that Bay 11-7082 (Figure 6B), and NBD binding peptide (Figure 6C) have a significant inhibitory effect on Egr1 induction when compared to control (Figure 6A). These results demonstrated that NFκB participated in the induction of Egr1 upon infection.

## Discussion

Egr-1 is involved in the gene expression and the replication of several viruses such as Epstein Barr virus [31,32], HSV-1 [17,33,34], JC virus [35], orthopox virus [36], murine corona virus [37], rabies virus [38,39], borna disease virus [39], human foamy virus [40], Japanese encephalitis virus [38], HTLV-I [41,42] and HTLV-II [42]. In most cases, Egr-1 was induced after the virus targeted neural cells and lymphocytes. Our results have demonstrated an induction of Egr-1 protein upon HSV-1 infection in epithelial cells such as VERO and SIRC (a corneal cell line). This observation suggests that Egr-1 could play a role in host cellular responses to HSV-1 lytic infection.

Several pathways are known to regulate the induction of Egr-1 including p38/MAPK [43], JNK [44], MEK/ERK [45], CREB pathway [46], and NFкB activation [27,28]. By inhibiting the pathways known to be involved in Egr1 induction, we determined that NFкB and CREB play roles in Egr1 expression induced by HSV-1. CREB is activated and stabilized by HSV-1 ICP10 and can modulate viral-induced apoptosis [47]. Studies have reported the presence of NFкB regulatory sequence [24] and CRE [25] in the human Egr-1 promoter. Transient activation of NFкB during the first few minutes of HSV-1 infection has been reported [48,49]. In addition, most of the up-regulated mRNA upon infection was NFкB-dependent [50]. Furthermore, NFкB-dependent gene expression is directly related to a number of stress-induced activities in eukaryotic cells [51]. Since Egr-1 is induced by stress and is known to control a variety of divergent cellular responses, it is possible that this induction is NFкB-dependent. Therefore from the host cell standpoint, viral infection can be considered as a stress and Egr1 is rapidly induced to turn on various cellular responses.

Our previous study showed that Egr1 can bind to the Egr1 binding element (EBE) located in the intron of ICP22 and thus regulate the activity of both ICP22 and ICP4 [17]. Since Egr1 is expressed in neural tissues, it could play a role in the maintenance of latency and subsequent reactivation. In addition, our preliminary studies revealed that Egr1 induced by viral infection was sufficient to interact with the ICP22 EBE during the lytic infection of SIRC and the over-expression of Egr-1 enhanced HSV-1 gene expression, replication, and release of infectious viruses (unpublished data). Together, these results suggest the involvement of Egr-1 in HSV-1 gene expression/replication via regulation of α-genes. Recombinant virus over-expressing Egr-1 and stable cell lines containing siRNA repressing Egr-1 are being constructed to further investigate the roles of Egr-1 during HSV-1 lytic infections.

It is not known which viral proteins are required for Egr-1 induction. It is likely that the binding of the viruses to the cell surface is sufficient to trigger the expression of Egr-1. Viral binding to the target cell is mediated by envelope glycoprotein gB, gC, or gD and the entry is mediated by gD to one of the cell surface receptors, such as the Herpes Virus Entry Mediator (HVEM)), heparan sulfate, nectin-1/2, and cell adhesion molecules from the immunoglobulin superfamily [52]. HVEM belongs to the superfamily of TNF (tumor necrosis factor)/NGF (nerve growth factor) receptors [53], and has been identified independently as TR2 (TNF receptor like-2) [54]. The Egr-1 gene can be regulated by TNF [55] and NGF [56] in cultured cells, and NFкB can be activated by the gD/HVEM [57]. Therefore, Egr-1 induction quite possibly can be correlated to the binding of viral glycoprotein to HVEM or other cellular surface receptors. Additional experiments are required to determine the induction mechanisms of Egr-1 by viral infection.

## Conclusion

In summary, we have shown for the first time that Egr1 can be induced rapidly by HSV-1 lytic infection in epithelial cells such as VERO and SIRC. This induction may be due to transactivation by the NFкB and/or CREB-mediated signaling cascades. The Egr1 induced by HSV-1 in corneal cells could have implications in viral pathogenesis, inflammation, and disease progression. More experiments to determine the role of Egr-1 are underway, especially in other models of infection.

## List of Abbreviations

ACV: acyclovir; ChIP: chromatin immunoprecipitation; CREB: cAMP response element binding protein; DAPI: 4',6-diamidino-2-phenylindole; DMEM: Dulbecco's modified Eagles medium; EBE: Egr1 binding element; EGR: Early growth response; FBS: fetal bovine serum; GFP: green fluorescent protein; HVEM: herpes virus entry mediator; NBD: NEMO-binding domain; NGF: nerve growth factor; PBS: phosphate buffered saline; RIPA: radioimmunoprecipitation assay; SIRC: Statens Seruminstitut Rabbit Cornea; TNF: tumor necrosis factor; TR2: TNF-receptor like-2; VERO: derived from kidney epithelial cells of the African green monkey.

## Competing interests

The authors declare that they have no competing interests.

## Authors' contributions

GRB established the experimental protocols, performed the infection and immunofluorescent microscopy, completed the Western blot analyses, composed the draft of the manuscript, etc. JRP performed the RT-PCR, assisted the preparation of manuscript, performed the transfection experiments, confirmed the RT-PCR results, and maintained the cell cultures. LG performed the RT-PCR, Western blot analysis and repeated the transfection experiments. HEM and JMH participated in the experimental design, prepared the expression vectors, discussed the experimental data, conceived the strategic plan, and participated in the manuscript preparation. SVH initiated the project, identified the Egr-1 induction, directed all the experimental approaches, analyzed the preliminary data, supervised the work, and prepared the manuscript. All authors read and approved the final manuscript.
